# A mitochondria cluster at the proximal axon initial segment controls axodendritic TAU trafficking in rodent primary and human iPSC-derived neurons

**DOI:** 10.1007/s00018-022-04150-3

**Published:** 2022-02-04

**Authors:** Noah Tjiang, Hans Zempel

**Affiliations:** 1 Institute of Human Genetics, Faculty of Medicine, University Hospital Cologne, University of Cologne, 50931 Cologne, Germany; 2 Center for Molecular Medicine Cologne (CMMC), Faculty of Medicine, University Hospital Cologne, University of Cologne, 50931 Cologne, Germany

**Keywords:** Axon initial segment/AIS, Mitochondria, TAU, Live-cell-imaging, Alzheimer's disease, Neuron, Neuronal cell polarity, Microtubule, Tauopathy, Mitochondriopathy

## Abstract

Loss of neuronal polarity and missorting of the axonal microtubule-associated-protein TAU are hallmarks of Alzheimer’s disease (AD) and related tauopathies. Impairment of mitochondrial function is causative for various mitochondriopathies, but the role of mitochondria in tauopathies and in axonal TAU-sorting is unclear. The axon-initial-segment (AIS) is vital for maintaining neuronal polarity, action potential generation, and—here important—TAU-sorting. Here, we investigate the role of mitochondria in the AIS for maintenance of TAU cellular polarity. Using not only global and local mitochondria impairment via inhibitors of the respiratory chain and a locally activatable protonophore/uncoupler, but also live-cell-imaging and photoconversion methods, we specifically tracked and selectively impaired mitochondria in the AIS in primary mouse and human iPSC-derived forebrain/cortical neurons, and assessed somatic presence of TAU. Global application of mitochondrial toxins efficiently induced tauopathy-like TAU-missorting, indicating involvement of mitochondria in TAU-polarity. Mitochondria show a biased distribution within the AIS, with a proximal cluster and relative absence in the central AIS. The mitochondria of this cluster are largely immobile and only sparsely participate in axonal mitochondria-trafficking. Locally constricted impairment of the AIS-mitochondria-cluster leads to detectable increases of somatic TAU, reminiscent of AD-like TAU-missorting. Mechanistically, mitochondrial impairment sufficient to induce TAU-missorting results in decreases of calcium oscillation but increases in baseline calcium, yet chelating intracellular calcium did not prevent mitochondrial impairment-induced TAU-missorting. Stabilizing microtubules via taxol prevented TAU-missorting, hinting towards a role for impaired microtubule dynamics in mitochondrial-dysfunction-induced TAU-missorting. We provide evidence that the mitochondrial distribution within the proximal axon is biased towards the proximal AIS and that proper function of this newly described mitochondrial cluster may be essential for the maintenance of TAU polarity. Mitochondrial impairment may be an upstream event in and therapeutic target for AD/tauopathy.

**Supplementary Information:**

The online version contains supplementary material available at 10.1007/s00018-022-04150-3.

## Introduction

Alzheimer’s disease (AD) and other tauopathies are neurodegenerative disorders that impose a huge burden on the aging society [1]. Pathological hallmarks of these tauopathies are abnormal phosphorylation, subcellular mislocalization and eventually, aggregate formation (mainly neurofibrillary tangles) of the microtubule-associated protein TAU [2]. Understanding the detrimental cascade of these TAU alterations is crucial for developing effective therapies and prevention options. However, the underlying mechanisms remain largely elusive.

In healthy neurons, TAU is sorted into the axonal compartment but in disease conditions, this axonal targeting of TAU fails, and the subsequent somatodendritic accumulation of TAU results in various pathological effects, ranging from protein-aggregation-based sequestering of various proteins to the mislocalization and aberrant activation of other proteins like FYN or TTLL6. Furthermore, TAU missorting is associated with loss of microtubules and mitochondria, all of which leads to neuronal damage, neuronal dysfunction and ultimately neurodegeneration [2]. While the mechanisms of axonal TAU sorting are still under debate [3, 4], there is evidence for a major role of the axon initial segment (AIS) for mediation of axonal TAU targeting [5, 6]. The AIS is a 20–60 µm long segment at the proximal axon, and the site of action potential (AP) generation [7]. The AIS is characterized by a highly structured cytoskeleton with enrichment of specific proteins (e.g. ANKRYIN-G, TRIM-46), very particular microtubule structures (highly structured fascicles) and dynamics (highly unstable microtubules, but high density and possibly EB3-mediated tethering to membranes) [5, 8–10] and voltage gated potassium/sodium channels [11].

Multiple studies have shown that the knockdown of AIS components (e.g. ANKYRIN-G) results in mistrafficking of TAU, implicating the relevance of an intact AIS for the maintenance of neuronal health and polarity [5, 9]. Studies with rodent primary neurons demonstrated that the AIS composes a TAU-diffusion barrier (TDB) [5, 10, 12] that controls retro- and anterograde trafficking of TAU. The mechanisms behind this TDB are largely enigmatic.

Besides losing proper axonal sorting of TAU, tauopathies and models thereof are associated with defects in mitochondrial function [13, 14]. Normally, mitochondria fulfill crucial tasks such as the homeostasis of calcium, of reactive oxygen species (ROS), taking part in various metabolic processes, and—most importantly—the generation of ATP. Mitochondria are steadily recycled via mitophagy and biogenesis, which in neurons mainly takes place in the soma [15]. The disease cascades of tauopathies lead to alterations of the mitochondrial homeostasis, e.g. an increase of fission proteins such as dynamin-related protein 1 (DRP1) [16] which may lead to excessive production of ROS and altered mitochondrial trafficking [17]. This disrupts the (re-)distribution of mitochondria into the meter-long neurites which in turn intensifies the decline of neuronal health in these diseases [18, 19].

When modelling the contributions of mitochondria as disease meditators, *global* mitochondrial impairment through external factors or genetic mutations alone is sufficient to achieve tauopathy-like neurodegeneration like TAU-hyperphosphorylation and TAU-missorting both in vitro and in vivo [20]. The *local* effects of mitochondria and their potential dysfunction, especially in areas that are crucial for efficient TAU sorting—like the AIS—however, are unstudied.

In this study, we describe a mitochondrial cluster in the proximal AIS present in murine primary cortical neurons as well as in human iPSC-derived cortical neurons, which influences axonal TAU sorting. The mitochondria of this AIS-cluster participate in fewer trafficking events and are less mobile compared to other axonal mitochondria. By inducing mitochondrial impairment, both globally but also specifically in this cluster, we could demonstrate that efficient TAU sorting relies on the proper function of these specific mitochondria. Mechanistically, shifting microtubule dynamics to more stable microtubules prevented mitochondrial impairment-induced TAU missorting, but chelating intracellular calcium did not, despite the fact that mitochondrial impairment led to increased cytosolic calcium. Our results suggest a key role of AIS-located mitochondria for the maintenance of TAU cellular polarity and give insights into disease mechanism of AD and other tauopathies.

## Materials and methods

### Molecular biology

To obtain a photoconvertible mitochondrial marker, we replaced the fluorescent protein sequence of mito-RFP with a sequence encoding Dendra2c via restriction cloning using SalI and BglII. To avoid spectral overlap in the live imaging experiments, a far-red fluorescent edition of the AIS-marker YFP-Na_V_-II-III (a gift from Matthew Grubb; Addgene plasmid #26056) was created by cloning the NaV-sequence into the vector of emiRFP670 (a gift from Vladislav Verkhusha; Addgene plasmid #136556) using NotI, SacI and mutagenesis-PCR.

### Cell culture and immunostaining

All experiments involving animals were conducted according to local legislation and animal welfare guidelines. Mouse primary neurons (MPNs) were prepared as described and characterized [5, 21]. Briefly, forebrain neurons were isolated by dissecting embryonic day 13.5 FVB/N mice and isolating the cortical hemispheres after extraction of the brain. These were digested with 1 × Trypsin (Panbiotech) and diluted in neuronal plating medium (Neurobasal medium, 1% FBS, 1 × Antibiotic/Antimycotic Solution; ThermoFisherScientific (TFS), 1 × NS21 (Panbiotech)), plated in in a either 24-well-plate (VWR) onto coverslips pre-treated with poly-d-lysine (PDL; Sigma-Aldrich) or a PDL-treated “IBIDI-µ-Dish 35 mm, high Grid-500 Glass Bottom” for live imaging/re-locating cells and then kept in a humidified incubator at 37 °C and 5% CO_2_. After four days, the same amount of neuronal maintenance medium (Neurobasal media (TFS), 1 × Antibiotic-/Antimycotic solution (TFS), 1 × NS21 (Panbiotech) and AraC (Sigma-Aldrich)) was added and cells were kept in culture as long as indicated. Human WTC11 iPSCs carrying a doxycycline-inducible Neurogenin2 (Ngn2) transgene were differentiated into neurons as previously described and characterized [22, 23].

MPNs and iPSC-derived neurons were transfected according to the manufacturers’ protocol with PolyJet (tebu-bio/Signagen) and Lipofectamine Stem Transfection Reagent (TFS), respectively, and plasmids were expressed for the indicated time period.

Cells were fixed and stained as previously described [21]. In brief, they were fixed with 3.7% formaldehyde (Sigma-Aldrich; in PBS), blocking and permeabilization were achieved by incubating for 5 min with 0.5% Triton-X and 10% bovine serum albumin in PBS. Primary antibodies were incubated overnight at 4 °C, secondary antibodies at RT for 1 h. After adding NucBlue (TFS) to stain nuclei, coverslips were mounted using AquaPolyMount (Polysciences). The following antibodies were used:

### Primary antibodies

**Table:** 

Mouse-anti-ANKYRIN-G	Neuromab/Biozol N106/36, #75-146	Monoclonal	1:250
Rabbit-anti-TRIM-46	Synaptic Systems, #37703	Polyclonal	1:500
Chicken-anti-MAP2	Abcam, #ab5392	Polyclonal	1:1000
Rabbit-anti-TAU	Dako, #A0024	Polyclonal	1:1000
Mouse-anti-MTCO1	Invitrogen, 1D6E1A8, #459600	Monoclonal	1:500
Rabbit-anti-ACETYL-TUBULIN	Cell Signaling, D20G3, #5335	Monoclonal	1:1000
Rat-anti-TYROSINATED-TUBULIN	Sigma-Aldrich, #MAB1864-I	Monoclonal	1:1000

### Secondary antibodies

**Table:** 

Donkey-anti-mouse 647	AlexaFluor (Invitrogen)	1:1000
Donkey-anti-chicken 647	AlexaFluor (Invitrogen)	1:1000
Donkey-anti-mouse 561	AlexaFluor (Invitrogen)	1:1000
Donkey-anti-rabbit 561	AlexaFluor (Invitrogen)	1:1000
Donkey-anti-rabbit 488	AlexaFluor (Invitrogen)	1:1000
Donkey-anti-rat 488	AlexaFluor (Invitrogen)	1:1000
Donkey-anti-Chicken CF488A IgY (H + L)	Linaris/Biotium, #20166	1:1000

Cells were treated as indicated with Rotenone or Antimycin A dissolved in Ethanol, diluted with cell culture medium; control cells were treated with an equal amount of carrier solution. For Fig. 4h, cells were treated with 10 nM Taxol (Sigma Aldrich), 5 µM BAPTA-AM (Sigma Aldrich) with or without 50 nM Antimycin A.

Somatodendritic TAU fluorescence intensity was measured in a semi-automatic way by detecting somatodendritic regions of interest (ROI’s) via MAP2, the mean background fluorescence intensity of the image was subtracted, and treated cells were normalised to control cells.

### Imaging and photoactivation/conversion

Epifluorescence microscopy was performed with a Zeiss axioscope equipped with Colibri 7 LED and an Axiocam 503 mono. Preparation for STED-nanoscopy followed the recommendations from the manufacturer, imaging was conducted with a Leica gSTED TCS SP8 system using AntifadeGold mounting medium (Invitrogen). Confocal fluorescence, live-cell-imaging and photoconversion/photoactivation (see below) was done using a Perkin Elmer-Yokogawa CSU-X1-Spinning-Disk-Confocal-Microscope.

After identifying the AIS and the corresponding mitochondria (using the NaV-emiRFP-670), mito-Dendra in the selected ROI was converted with the 405 nm-laser of the integrated FRAP tool. Images were acquired in both green and red channels every 2–5 s as a three-layered z-stack (step size 1 µm) for 30 min. Non-suitable cells (e.g. no cotransfection or no mitochondrial movement) were excluded from analysis.

To find out the appropriate parameters for the successful photoactivation of *mito-photo-DNP* (MPD) in neuronal cells, we added the live-imaging mitochondrial-membrane-potential (ΔΨ) marker *tetramethyl-rhodamine-methyl-ester* (TMRM; 10 nM; TFS) to the culture and titrated the adequate laser powers and concentration of MPD where TMRM fluorescence and thus ΔΨ would significantly drop, indicating mitochondrial disruption. Subsequent experiments were carried out without TMRM using the established parameters.

Cells were transfected with mito-RFP and NaV-emiRFP-670 to identify the AIS and corresponding cluster, after selecting adequate cells and ROI’s, 200 nM of MPD (BioTrend, dissolved in DMSO (Carl Roth)) was added and after 20 min of incubation, photoactivation in the ROI was achieved with a 355 nm-Laser (Rapp-OptoElectronic). Cells were kept in the incubator after treatment for 1.5 h before fixing and staining for TAU. For control cells, MPD was either activated in the soma with a ROI of comparable size or the laser was used on the AIS-mitochondria-cluster before the addition of MPD. TAU fluorescence intensity was then measured in the soma of treated cells and normalized to that of untreated cells (Ctrl) in the same dish.

To image spontaneous Ca^2+^ oscillations, MPN were labelled with 2 µM Fluo-4 (TFS) and 0.2% Pluronic F127 (Merck Millipore) for 20 min and washed once before imaging. Time-lapse-imaging was done for one minute in set intervals with 1 frame per second. After the first baseline recording, either 50 nM AMA, 5 µM BAPTA-AM or vehicle (conditioned medium) was added to the live imaging chamber.

### Data analysis and statistics

Image analysis was done using Fiji-ImageJ [24], graphing and statistical analysis was performed using GraphPadPrism 8. Data were analysed for normal distribution and statistical tests were performed accordingly (for details on statistical tests, see corresponding figure legend).

Cluster prevalence assessment and comparative analysis was done by categorizing each valid cell using three criteria: (1) mitochondria present in the proximal AIS (range ± 7.5 µm); (2) relative absence of mitochondria in the central AIS; (3) visible accumulation/higher brightness of the mitochondria in the proximal AIS. Cells were then categorized into three groups: cluster (+): 3 criteria met; cluster (~): 2 criteria met; and cluster (–): ≤ 1 criterion. The ratio was calculated as $\mathrm{Ratio}=\frac{{n}_{\mathrm{Total}}}{{(n}_{(+)}+\mathrm{0,5} *{n}_{(\sim )})}.$

The mitochondrial distribution along the AIS was measured by drawing in a line from the soma through the axon and creating a plot profile of the intensity of ANKYRIN-G. This was smoothened using a 1 µm sliding-mean and the “starting point” of the AIS was set to the point where this sliding mean was first ≥ 30% of its maximum (adapted from Dumitrescu et al. [25]).

To account for the difference in fluorescence intensity between cells and the high background fluorescence near the soma, mito-RFP images were thresholded using Trainable Weka segmentation (Arganda-Carreras, 2017), plot profiles from these binary pictures were generated with the same line-ROI’s, fitted to the “starting point” of the AIS and then averaged over all cells. Because the ROI’s had differing lengths, *n* got lower towards the extremes, so these data were not used. To statistically test the difference in distribution, the mean of each replicate over a span of 10 µm was used and the distance between the two lines was kept the same in Fig. 2h and i.

While segmenting the images into binary pictures solved the problem of varying fluorescence intensity, this eliminated a property of the AIS-mitochondria cluster: most often it was brighter than comparable mitochondria in the more distal AIS, a feature that is not reflected by this quantification. To that end and as a surrogate for mitochondrial mass, the raw fluorescence intensity of mito-RFP was used in a similar fashion. To account for the high background intensity near the soma, it was plotted with the fluorescence intensity of td-Tomato, which was measured and fitted to the AIS-start as described above as well. The diameter of the AIS was measured using td-Tomato and measuring every 1 µm. Cross-sectional area was extrapolated from diameter using $Y={\left(\frac{x}{2}\right)}^{2}*0.6*\pi$.

Kymographs were created using the KymographBuilder plugin in Fiji-ImageJ. All trafficking events that went past the cluster were counted, categorized into red/green and antero-/retrograde and expressed as a fraction of each cells total trafficking events.

## Results

### Global mitochondrial impairment leads to deficient TAU-sorting

**Fig. 1:**
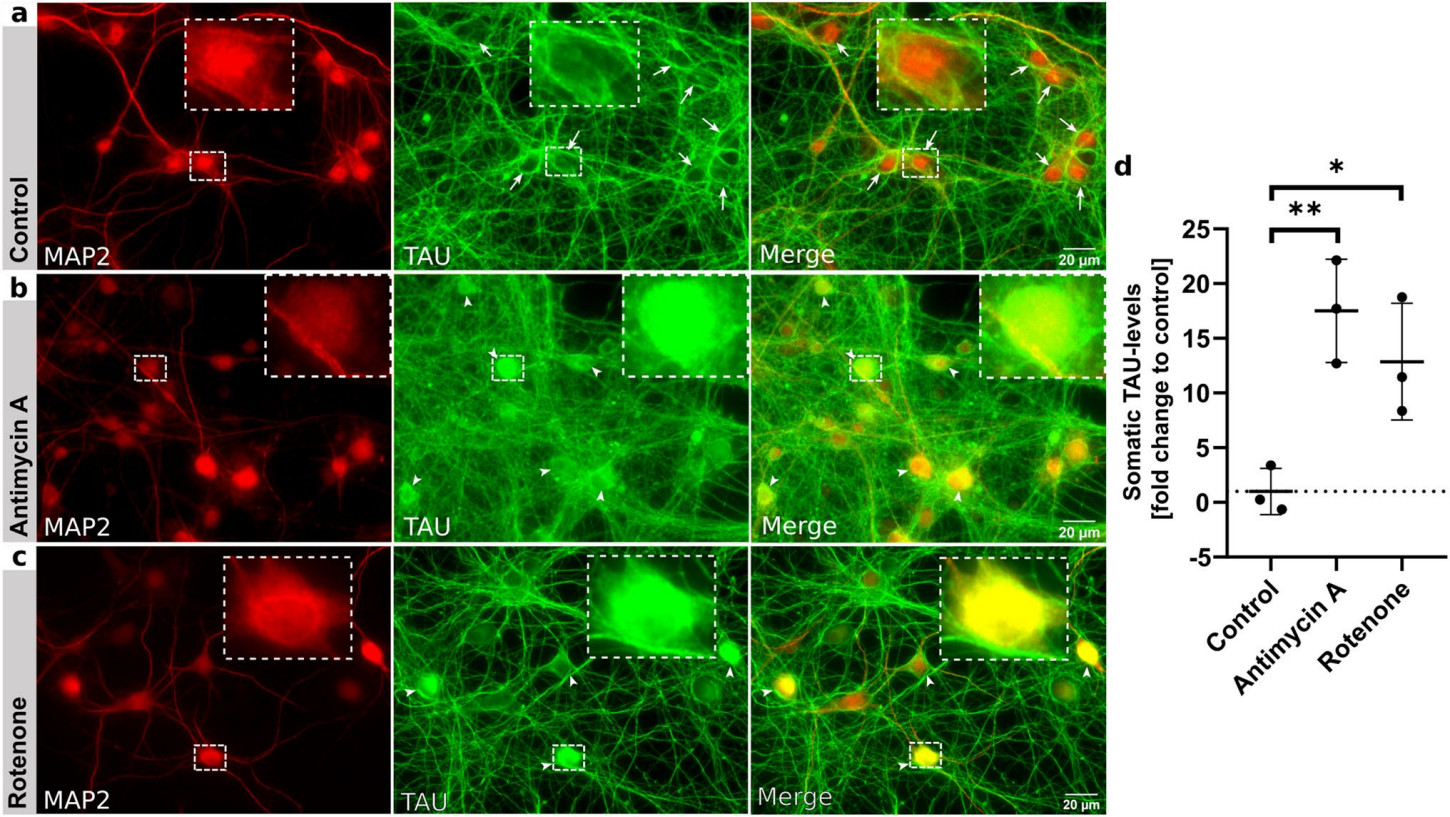
Global mitochondrial impairment leads to TAU missorting. **a–c** Immunofluorescence staining of murine primary neurons (DIV21) for MAP2 (somatodendritic marker) and TAU, treated for 2 h with **a** control, **b** 50 nM Antimycin A(AMA) or **c** 50 nM Rotenone (ROT, both respiratory chain inhibitors), representative epifluorescence images. Arrows in **a** indicate low level of somatic TAU in control cells, inserts show magnifications of exemplary cell bodies; arrowheads in (**b**, **c**) indicate missorted TAU in the soma after treatment, soma shows higher TAU-fluorescence-intensity, therefore merge appears yellowish to green. **d** Quantification of TAU-fluorescence-intensity in the soma after subtraction of background fluorescence, arithmetic mean of 3 biological replicates with standard deviation (SD). Control: Mean = 1.0; *n* = 122–126; AMA: Mean = 17.51; *n* = 42–172; ROT: Mean = 12.87; *n* = 144–154. Ordinary one-way ANOVA with Dunnett’s correction for multiple comparisons, Ctrl vs. AMA*:* **p = 0.0059, Ctrl vs. ROT*:* *p = 0.0258, significance level *p* < 0.05

Chronic exposure of neurons to mitochondrial toxins leads to TAU hyperphosphorylation and aggregation [12, 18]. The acute effect of mitochondrial impairment on TAU sorting, however, is unclear. We aimed to investigate TAU intracellular localization after mitochondrial impairment by using mitochondrial stressors. First, we treated mouse primary neurons (MPN; 21 days in vitro (DIV)) with the respiratory chain inhibitors Rotenone (ROT) and Antimycin A (AMA) for 2 h. We quantified the levels of endogenous TAU in the soma using MAP2 (microtubule-associated protein 2) as somatodendritic marker (Fig. 1a–c). We observed a substantial difference in somatodendritic TAU levels after exposure to the mitochondrial toxins, indicating a failure of effective axonal TAU sorting. This was most striking after treatment with 50 nM AMA (approx. 18-fold higher TAU signals in cells somata, 13-fold for Rotenone; Fig. 1d). Longer treatment duration and higher doses resulted in elevated levels of cytotoxicity and cell death (data not shown). Altogether, these results show that an acute, global inhibition of mitochondrial function can induce profound somatodendritic TAU missorting, outclassing previously reported AD-like missorting induced by Amyloid-*β* by a factor of 5–10 (e.g. [26]).

### A mitochondrial cluster is localized at the proximal axon initial segment

**Fig. 2:**
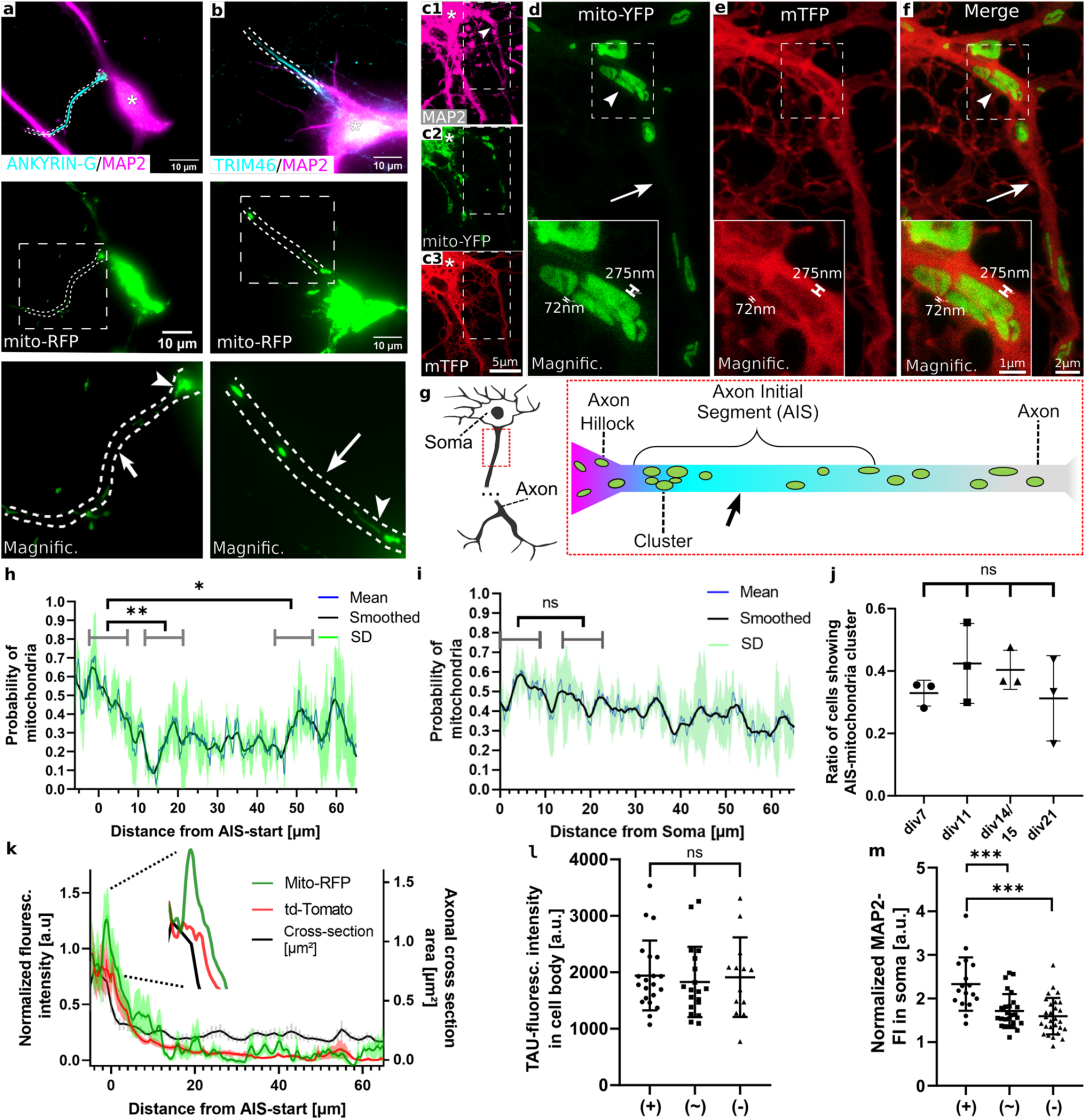
Mitochondria cluster at the proximal AIS in cortical rodent and human neurons. **a**, **b**: Representative images of DIV11 MPN (**a**) and DIV18 hiPSC-derived neurons (**b**) that were transfected with mito-RFP and immunostained for MAP2 (somatodendritic marker, magenta) and ANKYRIN-G/TRIM-46 (AIS-marker, cyan): Asterisks indicate the cell body, dashed lines indicate the AIS where MAP2 enrichment stops and AIS-markers are present; mitochondrial distribution magnified in lower row: arrowhead shows proximal cluster of mitochondria in the AIS, arrow indicates the region of lower mitochondrial abundance in the central AIS. **c**–**f** STED nanoscopy pictures of rat primary cortical neurons. Neurons aged 8-9DIV were transfected with mitoYFP (green) and mTFP (as a volume marker, red) for 2d, fixed and processed for two-colour STED nanoscopy. Arrow in (**c**) indicates beginning of the AIS (by decreasing intensity of MAP2, **c1**), dashed lines indicate magnifications in inserts in **d**–**f**. **d–f** Magnification of the axon, arrowhead shows proximal cluster of mitochondria, arrow shows subsequent region of lower mitochondrial abundance in the central AIS. Inserts show second magnification of the proximal AIS. Measurement of the shortest distance between the mitochondria and cell border (by mTFP) shown on either side shown in “Magnific.” **g** Scheme of a neuron, red box shows enlarged section of the proximal axon shown on the right: schematic of the observed mitochondrial distribution along the proximal axon, colouring indicates levels of MAP2 (magenta) and ANKYRIN-G (cyan) enrichment at the transition from soma to axon, cluster of mitochondria in the proximal AIS followed by a region of low mitochondrial abundance in the central AIS (marked by arrow). **h** Quantification of mitochondrial distribution along the AIS by segmentation. MPN transfected with mito-RFP on DIV8, fixed after 3d, and immunostained for ANKRYIN-G. Probability of a presence of mitochondria as a fraction, biol. replicate weighted mean (blue), standard deviation (green) and ~ 1 µm walking average (black). A region of higher mitochondrial abundance (cluster, proximal AIS) is clearly distinguishable from a region of low mitochondrial presence (central AIS). Mean_Cluster_ = 0.53; Mean_Centre_ = 0.22; Mean_Distal_ = 0.33; Brown-Forsythe and Welch’s ANOVA with Dunett’s correction for multiple comparison: cluster vs centre: **p = 0.0079; cluster vs distal: *p = 0.015; 3 biological replicates with *n* = 11–16, (for details, see “Methods”). **i** Similar procedure to (**h**) but measured over randomly selected dendrites as a control. Probability of a presence of mitochondria as a fraction, biol. replicate weighted mean (blue), standard deviation (green) and ~ 1 µm walking average (black). Mean_Start_ = 0.50; Mean_Centre_ = 0.42; unpaired *t*-test with Welch’s correction: not significant, *p* = 0.1278, 3 biological replicates with *n* = 17–20. **j** Ratio of cells showing the cluster morphology at different points in development, MPN were transfected with mito-RFP, fixed, stained for ANKYRIN-G, and classified according the appearance of the cluster (more details see “Methods”). Mean with SD, ordinary one-way ANOVA with Tukey’s correction for multiple comparisons: not significant, 3 biological replicates each, n for each replicate: *n*_div7_ > 21, *n*_div11_ > 18, *n*_div14_ > 15, *n*_div21_ > 5. **k** Quantification of mitochondrial distribution and corresponding axon width along the AIS. MPN were transfected on DIV8 with either mito-RFP or td-Tomato, an evenly distributed volume marker, fixed after 3d and immunostained for ANKYRIN-G. Axonal width was estimated by red: td-Tomato intensity along the AIS and black: extrapolation from the diameter that was measured every 1 µm (right y-axis). Insert shows a peak of mito-RFP-intensity indicating mitochondrial mass while the axonal width steadily declines. Fluoresc. intensity normalized so the first point equals 1. Mean with SEM, data from 3 biological replicates, *n* > 15. **l** Somatic TAU levels, classified by presence of AIS-mitochondria-cluster, for details see methods. Mito-RFP transfected MPN (DIV11) were fixed and immunostained for ANKYRIN-G and TAU. Mean with SD, Mean (+) = 1943, Mean (~) = 1829, Mean (–) = 1913, Ordinary one-way ANOVA with Tukey’s correction for multiple comparisons: not significant; 3 biological replicates with *n* > 15. **m** Similiar procedure as in (**l**), but immunostaining for MAP2. Somatic fluorescence intensity normalized by mean dendritic intensity. Mean with SD. Ordinary one-way-ANOVA with Tukey’s correction for multiple comparisons: (+) vs. (~) ***, (+) vs. (–) ***, (~) vs. (–) ns. Mean (+) = 2.333, Mean (~) = 1,714, Mean (–) = 1.595. 3 biological replicates with *n* > 17

As both the AIS [5, 6] and mitochondrial function (Fig. 1) are crucial for proper TAU sorting, we examined mitochondrial localization and dynamics in the AIS. First, we transfected both MPN’s (Fig. 2a) and hiPSC-derived-neurons (Fig. 2b) with mito-RFP (a mitochondrially targeted fluorescent protein), immunostained for the AIS-proteins ANKYRIN-G/TRIM46 to outline the AIS-position and measured the mitochondrial distribution along the AIS. This was done at four different timepoints after the initial establishment of neuronal cell polarity (meaning past “Banker stage” IV) [27].

In approx. 40% of the analysed neurons, we observed a characteristic pattern of mitochondrial distribution in the AIS. It consists of a cluster-like aggregation of mitochondria in the proximal AIS (arrowheads, Fig. 2a, b) and a subsequent zone in the central AIS that showed a lower abundance of mitochondria (arrows, Fig. 2a, b). This “AIS-mitochondria-cluster” coincided with the beginning of the AIS within a range of ~ 7.5 µm. We found this distinct mitochondrial distribution in both MPN and in hiPSC-derived neurons (Fig. 2a, b).

We further validated our initial observation of an AIS-specific mitochondrial cluster using two-colour STED super resolution microscopy. We used mito-YFP and the (presumably uniformly distributed) volume marker mTFP in superresolution mode (Fig. 2c1–c3). We confirmed the mitochondrial clustering in the proximal AIS and observed that the AIS-mitochondria-cluster is relatively large, leaving only a space of 70–300 nm to the sides. The mitochondria-cluster is not composed of a single, large mitochondrion, but rather several clearly distinguishable mitochondria (Fig. 2d–f; for a schematic depiction of the mitochondrial distribution within the AIS see Fig. 2g).

We next quantified and averaged the mitochondrial distribution along the AIS in MPNs and determined that the local mitochondrial prevalence was up to ten times higher in the proximal part of the AIS than in the central part. Even when averaged over a span of 10 µm, the prevalence was still about a 2.5-fold higher (Fig. 2h). Similar measurements along dendrites resulted in a mitochondrial presence probability as described before [28], but showed no significant differences in mitochondrial distribution along the dendrite (Fig. 2i), demonstrating that this biased mitochondrial distribution is a property unique to the AIS. There was no statistically significant difference between the proximal AIS and the proximal dendrite. As mitochondrial prevalence in a given neuritic segment does not reflect mitochondrial mass, we next determined the relative mitochondrial mass by measuring the fluorescence intensity of mito-RFP and compared this to the relative intensity of a volume marker (a FP presumably without a distribution bias, td-Tomato). In agreement with our prevalence measurements, we found that approx. 10 µm around the proximal border of the AIS, there was a pronounced discrepancy between the volume/diameter measurements and mitochondrial intensity indicative of mitochondrial mass, with mitochondrial signal being unproportionally high in the proximal AIS, but low to average within the central part of the AIS (Fig. 2k). In sum, mitochondria cluster in the proximal part of the AIS at least in a subset of neurons. Next, we examined the occurrence of this AIS-mitochondria at different time points of the neuronal maturation. For this, we transfected and fixed MPN at different days in vitro (Fig. 2j). The mitochondrial cluster was observable throughout our examined time course, with ~ 33% of neurons displaying it already after 7 DIVs and an increase in the proportion of AIS-mitocluster positive neurons to 43% in older neurons, although these results did not reach statistical significance.

As we observed the AIS-mitocluster consistently only in a subset of cells, and the AIS is a crucial regulator of neuronal cellular polarity, we next asked whether neuronal cellular polarity markers differ in neurons depending on the presence of the AIS-mitocluster. TAU and MAP2 are classical axonal and dendritic markers; low levels of TAU in the soma (and presence of TAU in the axon) hint towards well established neuronal cell polarity, while MAP2 shows ubiquitous presence both in soma and dendrites. We found no difference in somatic levels of TAU in neurons with clear presence of an AIS-mitocluster, compared to neurons without an apparent mitocluster or with an AIS-mitocluster that did not fulfill all of our criteria (Fig. 2l). Surprisingly, somatic and dendritic MAP2 levels were slightly, but statistically significantly higher in cells with a clear mitocluster in the AIS (Fig. 2m). This indicates that the pronounced presence of the AIS-mitocluster may be linked to at least some parameters of (MAP) cell polarity, although—at least in basal conditions—not for TAU.

### Mitochondria within the AIS-mitochondria-cluster are immobile

**Fig. 3:**
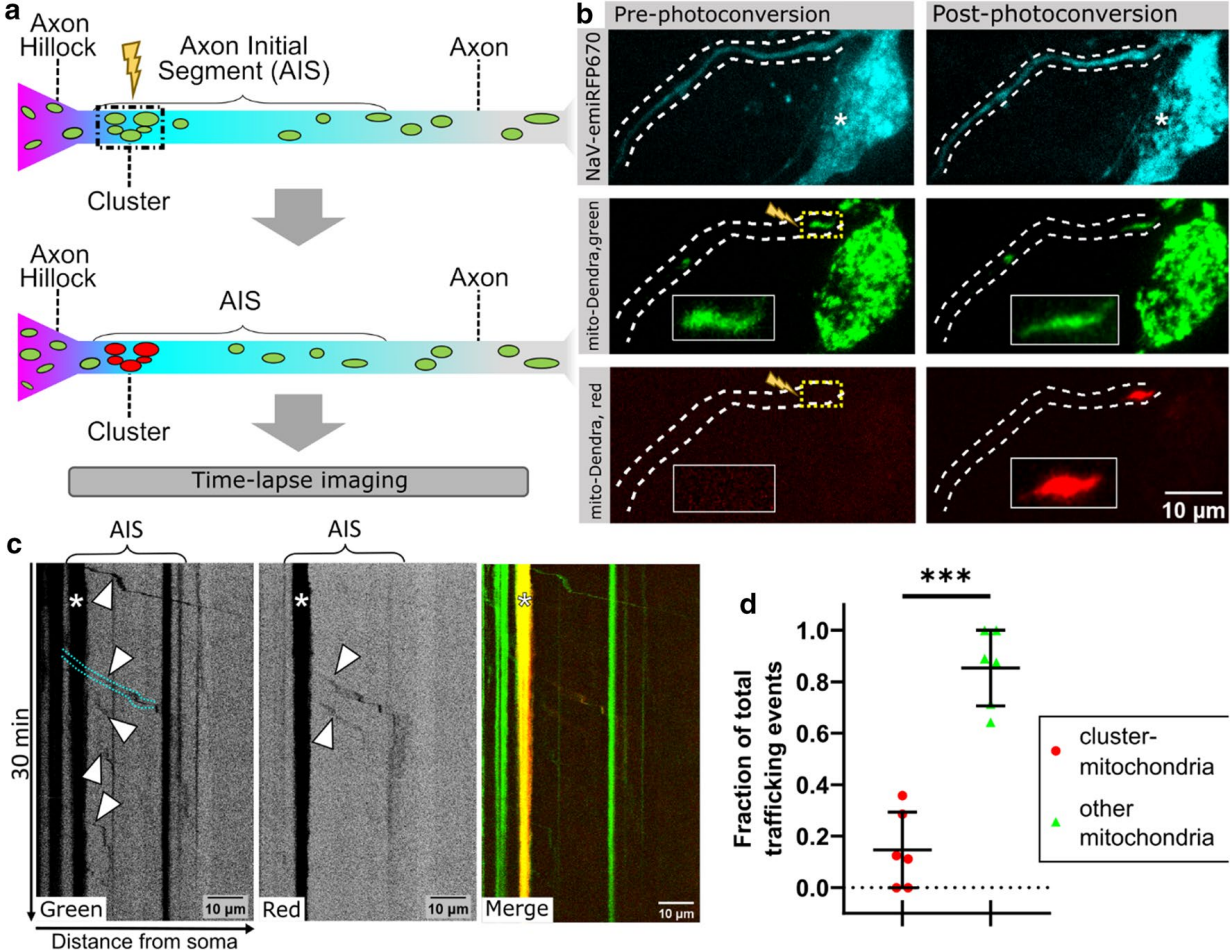
The mitochondrial AIS-cluster is immobile and only sparsely participates in axonal transport of mitochondria. **a** Scheme of the experimental workflow; after transfection with a photoconvertible mitochondrial marker (mito-Dendra) only the mitochondria of the cluster are converted from green to red by 455 nm laser illumination (boxed area, photoconversion indicated by yellow flash), while mitochondria in other localisations remained green. Cells were subsequently imaged for 30 min. **b** Primary cortical neurons (DIV7) were co-transfected with: NaV-emiRFP670 (top row), a live-cell-AIS-marker linked to a near-infrared FP and mito-DENDRA, a mitochondrial targeted green-to-red-photoconvertible FP. Middle shows green channel, bottom row red channel; before (left) and after (right) local photoconversion of the mitochondria in the cluster (enlarged in inserts and indicated by lightning bolt). Dashed line indicates the AIS, asterisks indicates the cell body. **c** Representative kymograph of the time-lapse imaging. Arrowheads show events of anterograde mitochondrial transport, cyan outline shows trafficking through and past the cluster, asterisk shows the stationary cluster in the proximal AIS. **d** Quantification of mitochondrial trafficking events in the AIS, split into mitochondria of the proximal cluster (red) and other mitochondria (green), shown as a fraction of each cells total trafficking events, Mean_Cluster-Mito_ = 0.146; Mean_Other-Mito_ = 0.853; unpaired *t*-test: ***, p < 0.0001, 4 biological replicates and *n* = 6

As mitochondria show net anterograde trafficking into the axon and the mitochondria-cluster is composed of several mitochondria, we hypothesized that it serves as a spawning hub for generating axonally trafficked mitochondria. We hence examined the dynamics of the AIS-mitochondria-cluster by co-transfecting mito-Dendra (a photoconvertible fluorescent protein that we fused to a mitochondrial targeting sequence) and the AIS marker Na_V_-emiRFP670 [25] into MPN (DIV7), and assessed mitochondrial mobility after four days of expression. We identified the AIS (using Na_V_-emiRFP670) and the corresponding cluster (using mito-Dendra) in live-cell-microscopy, photoconverted the mitochondria within the AIS-mitochondria-cluster from green to red, and conducted time-lapse-imaging (Fig. 3a, b).

This approach allowed us to distinguish between AIS-located mitochondria (red Dendra2) and mitochondria originating from other cell compartments (green Dendra2). Interestingly, we did not observe a single event when the whole AIS-mitochondria-cluster moved or dissipated in all our imaging sessions. Moreover, we observed axonal transport of mitochondria from the soma that passed through the proximal cluster at multiple times, seemingly without affecting it at all (Fig. 3c, cyan lines). In total, trafficking that originated from the AIS-mitochondria cluster only made up ~ 15% of all trafficking events in the AIS, while the other 85% originated from mitochondria from the axon and/or soma (Fig. 3d). In line with previous findings [29], we also saw that anterograde trafficking of mitochondria was more frequent than retrograde (here: about ~ 40% more). Retrograde trafficking almost exclusively (~ 98%) originated from mitochondria further down the axon and not from the AIS-mitochondria-cluster. Overall, this indicates that the cluster of mitochondria is anchored at the proximal AIS and does not serve as a hub for the axonal trafficking of mitochondria.

### The AIS-mitochondria-cluster plays a functional role in the maintenance of axonal TAU sorting

Since (i) the mitochondrial cluster is localized right at the AIS—a region crucial for maintaining neuronal cell polarity and proper sorting of TAU [5, 6], and (ii) cell-wide inhibition of mitochondria leads to impairment of these processes (namely TAU-missorting in our model, see above), we next tested whether the cluster of mitochondria might be involved in maintaining TAU cellular polarity.

**Fig. 4:**
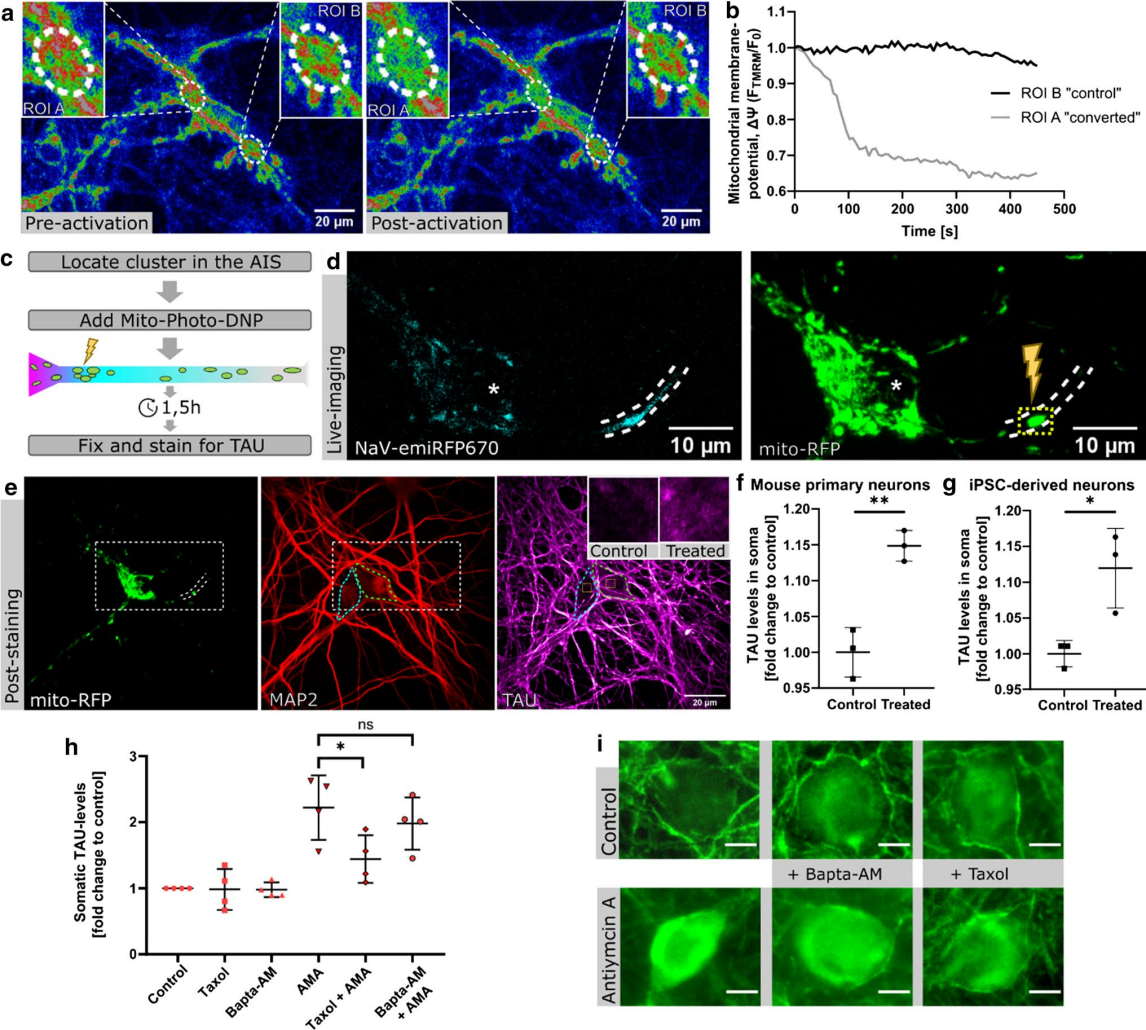
Local mitochondrial impairment of the AIS-mitochondria-cluster leads to TAU-missorting. **a** MPN (DIV11) were incubated with the mitochondrial membrane potential marker TMRM (10 nM) and the photoactivatable uncoupler Mito-PhotoDNP (MPD; 200 nM) for 20 min prior to photoactivation. MPD was only activated in “ROI A” by laser illumination, RGB-pseudocolouring of TMRM signal shows loss of mitochondrial membrane potential in the treated ROI A, but not in control ROI B. **b** Relative quantification of the mitochondrial membrane potential (ΔΨ) from the experiment in (**a**), graph shows the decline and persisting low level of TMRM-fluorescence-intensity that is limited to the photoactivated ROI A; time-lapse-imaging for 7.5 min, measured every 6 s to reduce photobleaching. **c** Scheme of the experimental workflow to achieve local mitochondrial impairment in the AIS in live cultured neurons with MPD and measure the effects on somatodendritic TAU levels. **d** Representative live-imaging pictures of the AIS and mitochondria in DIV11 MPN, lightning bold shows the mitochondrial cluster in the proximal AIS where mitochondrial impairment was induced (boxed area). Asterisks indicate cell body. **e** Representative confocal images of cells after photoactivation, fixation and immunostaining for MAP2 and TAU. White dashed lines indicate AIS, rectangle shows section from (**d**). Coloured dashed lines indicate the outline of the soma of the treated (green) and untreated cell (cyan). Magnifications show section of the soma (yellow boxes), note the slight elevation of TAU-fluorescence intensity in the soma of the cell that underwent photoactivation. **f**, **g** The experiment was repeated in 3 biological distinct replicates in both MPN and iPSC-derived-neurons, TAU-fluorescence intensity was measured in the soma of the treated cells (i.e. photoactivation of the mito-AIS-cluster). Untreated cells (i.e. also exposed to the MPD, but not photoactivated) in the same plate served as controls. **f** MPN, mean with SD, each point is one biological replicate; Control: Mean = 1.0, *n* = 5–16. Treated: Mean = 1.148, *n*_Total_ = 5; unpaired-*t*-test, **p = 0.0032. **g** iPSC-derived neurons, mean with SD, each point is one biological replicate, Control: Mean = 1.0, *n* = 6–17; Treated: Mean = 1.119, *n*_Total_ = 4; unpaired-*t*-test, *p = 0.0243. **h** Quantification of TAU-fluorescence-intensity in the soma after subtraction of background fluorescence, after treatment with either vehicle, 10 nM taxol or 5 µM BAPTA-AM ± 50 nM AMA for 2 h. Normalized as fold change relative to control. Arithmetic mean of 4 biological replicates (all n > 120) with SD. Mean_Taxol_ = 0.9826, Mean_BAPTA-AM_ = 0.9783, Mean_AMA_ = 2.20, Mean_Taxol+AMA_ = 1.441, Mean_BAPTA-AM+AMA_ = 1.980; Ordinary one-way ANOVA with Tukey’s correction for multiple comparisons: AMA vs. Taxol + AMA: *, *p* = 0.0325, AMA vs. BAPTA + AMA: ns. **i** Representative images of TAU fluorescence in the soma of MPN from the experiment in (**h**); somatic TAU is low in control and stable after treatment with taxol or BAPTA-AM alone (upper row); increase is visible in all AMA treated cells (bottom row), but taxol markedly prevents somatic TAU increase, scale bar 10 µm

We used the compound Mito-Photo-DNP (MPD) that allows precise, spatially and timely defined disruption of the mitochondrial membrane potential upon photoactivation in living cells, as previously reported [30]. To test our setup, we incubated MPN with 200 nM MPD and 10 nM TMRM, a live-imaging mitochondrial-membrane-potential marker. After photoactivation, mitochondrial membrane potential was strongly and stably reduced at the illuminated ROI (ROI A), while remaining unaffected in regions of the cell without photoactivation (ROI B, Fig. 4a, b).

Next, we tested the effect of local AIS-mitochondria-cluster impairment on sorting of TAU. We used DIV18 hiPSC-derived neurons and DIV11 MPNs (both of which show mature neuronal polarity and effective TAU sorting into the axon at this age) cotransfected with mito-RFP and Na_V_-emiRFP670 for 4 days (7 days for hiPSC-derived neurons) to detect the cluster within the neurons (Fig. 4c, d). We disrupted regional mitochondrial function by photoactivation of MPD at the AIS-mitochondria-cluster (Fig. 4c, d). After 1.5 h, cells were fixed and TAU levels in the soma were measured and compared to non-treated control cells via immunostaining (Fig. 4e).

A small but significant increase in somatodendritic TAU levels was observed after impairment of AIS-localized mitochondria (Fig. 4f, g) compared to untreated cells (~ 15% increase in MPNs and ~ 12% in iPSC-derived neurons). This effect is specific to axonal/AIS mitochondria since interference with somatic or dendritic mitochondria did not result in TAU missorting (Suppl. 2b). Additionally, irradiation of the cluster without MPD as a control did not result in any significant effect on TAU trafficking (Suppl. 2a), further proving the specificity of the treatment. Altogether, this indicates a crucial role of the small sub-population of mitochondria anchored in the proximal AIS for the maintenance of axonal sorting of TAU and may establish them as a vulnerable player in the complex disease cascades of tauopathies.

How does mitochondrial impairment lead to TAU missorting? The cause of TAU missorting/somatic accumulation of TAU in neurons both in human disease and in model systems is unresolved, but previous experiments clearly hint towards lack of anterograde routing of somatically synthesized TAU into the axon as the overarching cause [26]. Neuronal stress associated with increase in cytosolic calcium, induced by a.o. Amyloid-beta oligomers, glutamate, or simply high (~ two–five-fold) increases in extracellular calcium results in TAU missorting [31]. Also, in our previous studies, we consistently found that global loss of microtubules, or impaired microtubule dynamics in the AIS is associated with TAU missorting [5]. On the other hand, mitochondrial impairment (e.g. via respiratory chain inhibition) results in increased cytosolic calcium over time [32], and, as microtubules are highly dynamic particularly in the AIS, maintenance of labile and continuously rebuilding microtubule tracks in the AIS must also depend on ATP—and hence proper mitochondrial function. We thus tested whether calcium homeostasis is perturbed in our paradigms (rather low and non-cell-toxic concentrations of respiratory chain inhibitors) sufficient to induce TAU missorting. We consequently investigated cytosolic calcium levels via live-cell-imaging with a calcium-sensitive dye, Fluo-4, similar as before [22]. In agreement with previous experiments showing that mitochondrial impairment leads to increased cytosolic calcium levels (as calcium import into mitochondria requires a proper proton gradient, for review see [33]) and our initial findings that TAU missorting in response to mitochondrial impairment occurs within hours (Figs. 1, 4c–e), we found that 50 nM AMA treatment of primary neurons resulted in slight elevation of baseline calcium levels over the time of 1 h when assayed via Fluo-4 live-imaging (Suppl. 3a–c), to roughly twofold of initial baseline levels. Of note, this increase of overall cytosolic calcium was not due to increased calcium oscillation indicative of increased action potential generation/neuronal transmission; instead, we found that even though we used a low concentration, spontaneous activity of neurons was only stable in AMA-treated cells for roughly 20 min, before it continuously decreased until it ceased more or less completely after 40 min, while control neurons kept on firing/showed persistent calcium oscillation until at least 80 min in our conditions (Suppl. 3e, f). Thus, in agreement with previous data and with mitochondria being an important calcium storage/buffer organelle, calcium homeostasis is disturbed after mitochondrial impairment in our conditions, albeit to a rather small extent.

Next, as microtubule dynamics and/or stable microtubules are essential to maintain anterograde sorting of TAU and the TDB [12], we tested whether mitochondrial impairment would result in changed microtubule dynamics or more labile microtubules. We found that after mitochondrial impairment induced by AMA as before (50 nM, 2 h), both microtubule acetylation and tyrosination are increased (Suppl. 4). This shows that mitochondrial impairment leads to changed PTMs (hinting towards less de-acetylation and de-tyrosination) rather than to an active severing of microtubules (e.g. by the microtubule severing enzymes spastin, katanin, fidgetin), which are energy/ATP-dependent anyhow [34]. Next, we tested whether pharmacological manipulation of the two putative key triggers of TAU missorting, namely elevated cytosolic calcium or altered microtubule dynamics, could prevent the somatic TAU accumulation induced by mitochondrial impairment. To this end, we co-incubated neurons with an intracellular calcium-chelating agent, BAPTA-AM, or a microtubule-stabilizing drug, taxotere/taxol, at non-toxic but efficient concentrations (5 µM and 10 nM, respectively), together with AMA as above. Note that taxol is used at dramatically higher concentrations when used as an anti-cancer, anti-cell proliferation drug, while the concentrations we used here are sufficient to shift the microtubule-dynamics towards more stable microtubules, and prevent loss of microtubules in disease paradigms (see e.g. Zempel et al. [31]). The chosen BAPTA-AM concentration is adequate to induce a decrease in intracellular calcium (Suppl. 5), this way it should be sufficient to prevent or at least attenuate the part of TAU-missorting/neuronal damage that is triggered by an increase in calcium—while being subtle enough as to not have direct toxic effects at this timescale [35].

We found that taxol was able to significantly reduce the amount of AMA-induced TAU missorting by ~ 70%, while BAPTA-AM only slightly (by ~ 20%) and statistically non-significantly prevented TAU missorting. Together with the rather low levels of calcium increases in response to AMA treatment, this hints towards a role of impaired microtubule dynamics as the key mediator for mitochondrial impairment-induced TAU missorting.

## Discussion

In this study, we investigated the relation of mitochondrial (dys-)function and TAU (mis-)sorting. Our interest was triggered by the high amount of somatically mislocalized TAU in response to several respiratory chain inhibitors. We here employed two well established and well-researched specific mitochondrial toxins, in several concentrations and durations. Additionally, we used a photoactivatable, mitochondria-specific and mitochondrially targeted toxin (mito-PhotoDNP), which we activated in a spatially restricted manner. We are thus fairly confident that our interventions are specific for mitochondria. Of note, the amount of TAU missorting induced by mitochondrial impairment outclasses previously identified and studied triggers of TAU missorting, such as amyloid-β-oligomers (as a surrogate for AD-like stress), glutamate (excitotoxicity), and mutant TAU-overexpression (genetic tauopathy-like stress) [5, 12, 26, 36] by a factor of 5–10. Mitochondria play thus a crucial role for TAU neuronal cell polarity maintenance and might be important for disease. Similar to studies conducted before, mitochondrial impairment resulted in TAU missorting only under non-toxic conditions, in agreement with previous studies showing that protein synthesis must be functional for TAU mislocalization to occur [26]. In this study, we focussed on mitochondria within the AIS, as this is the currently suggested crucial site for proper TAU sorting and maintenance of neuronal cell polarity.

We discovered a characteristic pattern of mitochondrial distribution along the AIS, which consists of a proximal cluster of mitochondria and a region in the central AIS mostly devoid of mitochondria. The mitochondria of the cluster also frequently appear to be brighter than the average mitochondria along the axon, hinting towards a larger mitochondrial mass and number at that site. This is in line with our observations from STED-nanoscopy, which showed several densely packed mitochondria at the proximal AIS. We observed this cluster in both primary rodent forebrain as well as human iPSC-derived cortical neurons, indicating that this is a conserved feature across species and may be an important characteristic of the AIS—at least in cortical neurons. Interestingly, this AIS-mitochondria-cluster (as defined by the selected criteria, see Methods) was not observed in all the cells examined, but rather in about 30–45%. This fraction is roughly consistent over the development of the neurons, indicating that the AIS-mitochondria-cluster may be rather independent of neuronal maturation after the establishment of the AIS. Defined subpopulations of neurons that express this characteristic pattern of mitochondria seem plausible given that rodent forebrain cultures are still a heterogeneous mix of neurons when compared to our hiPSC-derived neurons [21]. On the other hand, our preliminary observations suggest that the AIS-mitochondria-cluster is not observable in all *Ngn2-*driven hiPSC-derived cortical neurons either, even though they are very homogenous [37]. This indicates that the mitochondria-cluster is observable only in a subset of primary but also homogenous clonal cells, thus the differences might stem from variable physiological characteristics of the AIS. These depend on the individual electrophysiological properties, differential network integration and varying places of origin of the AIS, but are also distinct in different subpopulation of neurons [38].

Other studies described similar clusters: one study investigated mitochondria in a limited number of chicken spinal motoneurons, and found similar to our study a biased distribution of mitochondria within the AIS [39]. Consistent with our data, they show that in the central part of the AIS, mitochondria are sparse, but found, in contrast to our data, that mitochondria accumulate only in the distal, but not in the proximal part of the AIS [39]. Another study, again in spinal motoneurons, observed that in ALS-transgenic mice expressing a human mutant SOD1, neurofilaments and (round-shaped) mitochondria are increasingly present in the AIS, but not in the soma, in disease conditions [40]. These studies do agree that it is likely that mitochondria accumulate, because there is a regions of high energy demand. In our case, the mitochondria may be clustered in the AIS to support the local generation of action potentials (AP). Another reason to why they are not evenly distributed along the AIS might be the varying distribution of different Na_V_ channels: Na_V_1.6 channels (responsible for AP generation) and high-threshold Na_V_1.2 channels are also enriched in two distinct groups along the AIS [41]. Future studies may investigate whether the AIS-mitochondria-cluster colocalizes with a specific subgroup of Na_V_ channels and whether they functionally interact.

We observed that the mitochondrial cluster is stationary at the proximal AIS as assessed via live-cell-imaging. The results from these experiments confirm the prior observation made in fixed cells and rule out that these observations were artifacts created by a zone of slower mitochondrial transport: Mitochondria still moved through the AIS while the cluster remained stationary, even when we tentatively increased imaging time up to several hours. In line with previous studies [42], exploratory experiments showed that photoconversion in itself did not cause reduced mitochondrial motility.

Our initial hypothesis—that the AIS-mitochondria-cluster might be a spawning hub for generating axonally trafficked mitochondria—was at least partially refuted since the AIS-mitochondria-cluster only sparsely participates in mitochondrial trafficking. There, the mismatch between the small number of photoconverted mitochondria in the AIS and relatively large number of non-photoconverted mitochondria in the soma could have falsely amplified our result, but given the small scale of both the observed area and relatively short timeframe, this bias should be small and not be able to cause the observed large difference in trafficking. Yet, the putatively AIS-mitocluster derived axonal mitochondria are overrepresented, as the AIS-mitocluster comprises far less than 15% of total cell mitochondria (rather around 0.1%, as estimated by mito-RFP-positive area). There are several interpretations: (1) As mitochondrial trafficking through the AIS is difficult (highly dynamic microtubules within the AIS, slow mito-trafficking [5]) soma-derived mitochondria might pause at the AIS-mitocluster long enough to be missed by our analysis, but eventually continue to the axon. (2) The primary function of the mitocluster is the supply of axons with mitochondria, but this is in contrast with our data, which clearly shows that the majority of axonally trafficked mitochondria are not AIS-mitocluster derived, and that many neurons with axonal mitochondria do not display a clearly discernible AIS-mitocluster. If the AIS-mitocluster would be the prime source of axonal mitochondria, continuous replenishment of the mitocluster itself would be necessary (to prevent accumulation of dysfunctional mitochondria/mtDNA), which we, however, in our experiments did not observe, but this could of course be due to a different timeframe in which these events take place. (3) The mitocluster has primarily other functions (e.g. local ATP-generation, calcium buffering), but may spawn mitochondria occasionally, while the reason for spawning might be specific (e.g. urgency) or statistic. Thus, it is possible that mitochondria travel out of the mitocluster into the axon, but spawning mitochondria is likely not the primary function of the AIS-mitocluster.

Nevertheless, it remains puzzling how mitochondria from the soma and/or axon pass the proximal cluster without affecting it, even though the space around the cluster appears to be very narrow (< 300 nm in STED-nanoscopy). A possible explanation would be that trafficking mitochondria follow the microtubule tracks in the centre of the axon, while the AIS-mitochondria-cluster could have a ring-like structure in the cross-section, allowing mitochondria to pass both ways. A former study using electron microscopy supports this hypothesis, showing that the mitochondria that “accumulate in the proximal segment of the axon […] occupy the peripheral regions of the axoplasm” [43], although this study was limited to rat hypoglossal nerve. This study and the other two studies mentioned before [39, 40] do not comment on the subcompartimental distribution of mitochondria, used different types of neurons, and also different methodology to define the AIS. Thus, it will be important to understand the cytoskeletal scaffold that might anchor the AIS-mitochondria-cluster in place. The overall mobility of membrane proteins is greatly reduced in the AIS, mainly because of the intertwined ankyrin-spectrin-actin-cytoskeleton [44, 45]. The AIS-mitochondria-cluster might interact with one of these AIS-specific scaffold proteins (e.g. actin via myosin) [46] or with syntaphilin-like proteins, which were found as a docking partner of axonal mitochondria in rat hippocampal neurons [47]. In-vivo-proximity-labelling of local interaction partners of the AIS-mitochondria-cluster could be a valuable experiment to investigate anchoring mechanisms.

Studying the functional aspects of the mitochondria-cluster in the proximal AIS, we found that local impairment of the AIS-mitochondria-cluster alone is sufficient to induce subtle, but detectable increase of somatic TAU protein. This TAU-missorting after local mitochondrial impairment was not as striking as the effect of global mitochondrial inhibition through ROT/AMA (see also Suppl. 1), but this difference was expected since the AIS-mitochondria-cluster makes up less than 0.1% of the cell’s total mitochondria (as estimated by mito-RFP-positive area).

In fact, these results suggest that the observed TAU-missorting after local impairment is an effect *specific* to the cluster-mitochondria, since they make up less than 0.1% of the cells mitochondria but lead to more than 1% of the missorting observed after global inhibition. This hypothesis is further supported by the fact that we did not observe measurable TAU-missorting when impairing a comparable small number of mitochondria in the somatodendritic compartment (Suppl. 2). Therefore, it is probable that the observed TAU-missorting after local impairment of AIS-localized mitochondria is due to a disruption of local processes in the AIS. One of these processes could be the constant rebuilding of microtubule tracks within the AIS. We have found previously that, while tubulin concentrations are high in the AIS, the amount of polymerized microtubules is rather low and there are no stable microtubules in the AIS, but the AIS may be a polymerization hub for microtubules [5, 48]. High-resolution scanning electron microscopy or STED-nanoscopy of AIS based microtubule requires the use of molecular densifiers and/or microtubule stabilizers to visualize microtubule tracks within the AIS [8]. All of this indicates that microtubules in the AIS are highly dynamic. Yet, all axonal cargo must pass through the AIS, and as axonal transport is predominantly microtubule based, the AIS-based microtubule-track must be continuously generated. The mechanisms responsible for the observed TAU-missorting may be (i) impaired anterograde sorting of TAU at the AIS, or (ii) disruption of the retrograde barrier that holds axonal TAU in place. On the anterograde side, the locally impaired mitochondrial homeostasis could impair the microtubule-based, anterograde transport of TAU-mRNA [49] and also the energy demanding, local TAU-protein-translation, proposed to mainly take place in the AIS [5]. We found that by stabilizing microtubules the AMA-induced TAU missorting was (partially) rescued. On the other hand, chelating calcium had no significant effect on the TAU missorting induced by mitochondrial impairment in our experiments. Although this mechanism of neuroprotection has been shown before for other neuronal injuries [50, 51], these injuries also led to a higher increase in calcium than our mitochondrial impairment which might be the cause that the protective effect was more pronounced there. Therefore, while both elevated calcium and microtubule impairment are tightly linked to TAU missorting and so far the two most discussed triggers [4, 52], in our case impaired microtubule function/dynamics may be causative or at least the major factor for mitochondrial impairment-induced TAU missorting.

On the side of the retrograde diffusion barrier failing, the locally reduced level of readily available energy providers (i.e. nucleoside triphosphates such as ATP) could also alter the functions of glycogen synthase-kinase-3β (GSK3β). GSK3β is a major kinase tethered in the AIS and is implicated with disease pathology in AD and dysfunction of the TDB [53, 54]. This theory is further supported by the finding that GSK3β is also activated by ROS [55]. It is very plausible that the local level of ROS is higher after our impairment of regional mitochondria. This would result in two different ways of how dysfunction of the AIS-mitochondria-cluster can lead to failure of the TAU diffusion barrier via alteration of GSK3ß. These two different functions of the AIS-mitochondria-cluster (energy and ROS homeostasis) have to be further investigated in follow-up studies.

All in all, we provide evidence for a cluster of mitochondria in the proximal AIS in different types of cultured neurons. This AIS-mitocluster is largely stationary and has a functional role in the maintenance of effective TAU sorting. These location-specific mitochondria may play a crucial role in the pathomechanisms of tauopathies such as Alzheimer’s disease and even in genetic mitochondriopathies. Thus, mitochondrial dysfunction may be upstream of TAU pathology in Alzheimer’s disease and related tauopathies, and may be a relevant therapeutic target in the future.

### Supplementary Information

Below is the link to the electronic supplementary material.

Supplementary file1 (DOCX 2624 KB)

## Data Availability

The original contributions presented in the study are included in the article/supplementary material, further inquiries can be directed to the corresponding author.

## Abbreviations

AD: Alzheimer’s disease
AMA: Antimycin A
AIS: Axon initial segment
AP: Action potential
FI: Fluorescence intensity
hiPSC: Human-induced-pluripotent stem cells
MPD: Mito-Photo-DNP
PTM: Post-translational modification
ROT: Rotenone
TDB: TAU-diffusion-barrier
TTLL6: Tubulin-Tyrosine-Ligase-Like-6
