# Supplementary material for: A mitochondria cluster at the proximal axon initial segment controls axodendritic TAU trafficking in rodent primary and human iPSC-derived neurons
Source: Cell Mol Life Sci. 2022 Feb 4;79(2):120. doi: 10.1007/s00018-022-04150-3 (PMC8816743; doi:10.1007/s00018-022-04150-3)
Supplement: Supplementary file 1 — Supplementary file1 (DOCX 2624 KB) [file 18_2022_4150_MOESM1_ESM.docx]

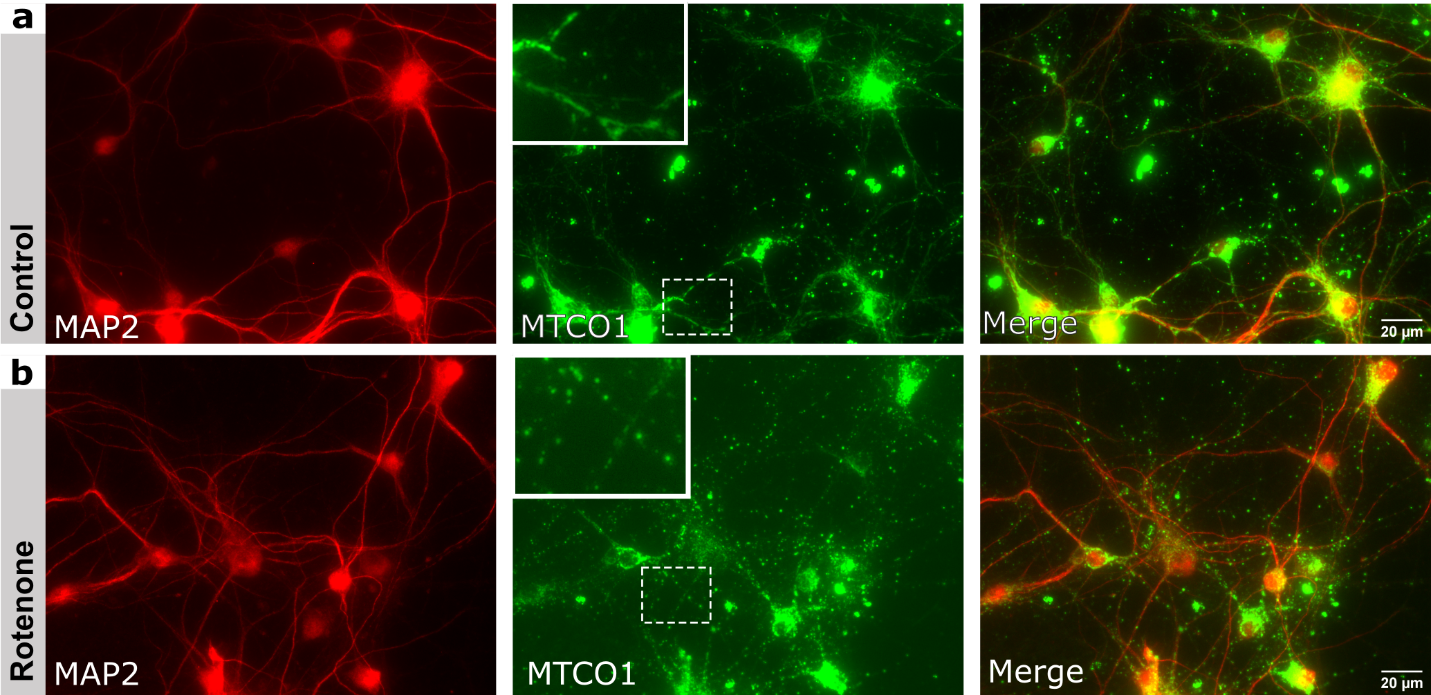


**Suppl. 1** **Respiratory chain inhibitors** **induce a global change of mitochondrial morphology**
Additional immunofluorescence staining of experiment from Fig. 1 for MAP2 and MTCO1 (mitochondrial marker).
**a**: Magnification shows healthy, elongated mitochondria forming a network.
**b**: Changed morphology of mitochondria after treatment with 50µM Rotenone for 2 hours, magnification shows scattered and rounded mitochondria as a sign of stress.


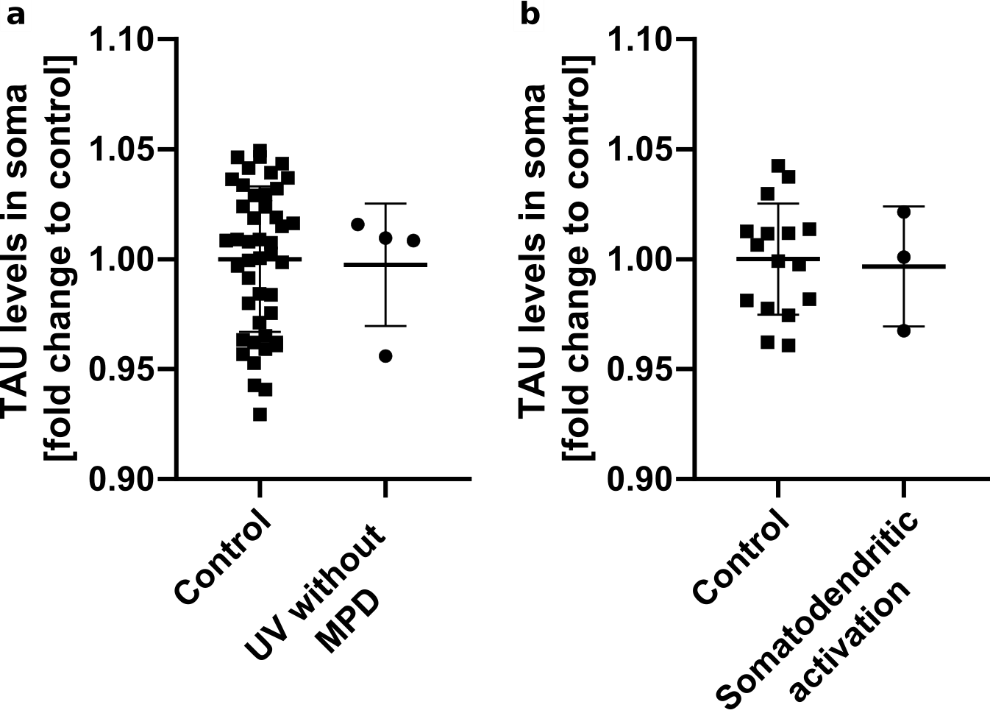


**Suppl. 2 Somatodendritic activation of MPD does not result in TAU-missorting**
**a-b**: The experiment from Fig. 4 e-g was also performed in co-transfected control cells in both MPN and iPSC-derived-neurons, this time firing the UV-laser at the AIS-mitochondria cluster before the addition on MPD (a) or at a ROI of similar size in the somatodendritic compartment after addition of MPD (b).
TAU-fluorescence intensity was measured in the soma of the these cells, untreated cells in the same plate served as controls (similar to Fig. 4f-g). Each point represents one cell, a: Mean_Control=_1.0, Mean_UV without MPD_=0.9974
b: Mean_Control_=1.0, Mean_Somato.act_=0.9966.


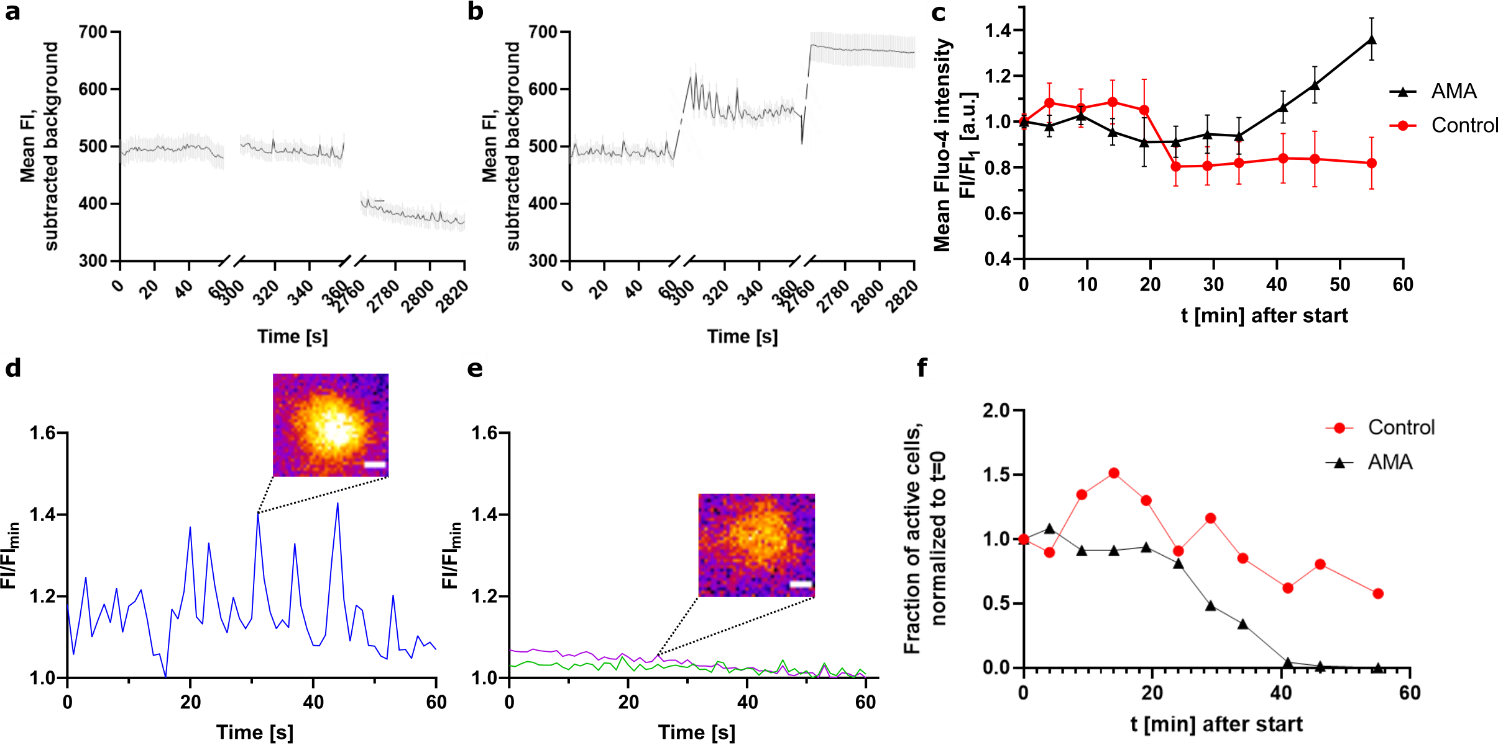


**Suppl. 3 Antimycin A induces increase of calcium levels in a short timeframe
a-b:** MPN (DIV11) were for prepared calcium imaging (see methods) and treated with vehicle (a) or 50nM AMA (b) and directly imaged. Mean fluorescence intensity and SEM of all cells in the field of view (n>90) with subtracted background  **c:** Overall change in mean FI normalized to first datapoint; control shows expected decline in FI due to bleaching, while AMA shows an inverse effect.
**d**: Exemplary graph of an actively oscillating neuron, insert shows calcium level at peak. FI normalized to minimal FI of that neuron.
**e**: Exemplary graph of an inactive neuron, insert shows basal state with no significant deviations.
**f**: Fraction of actively oscillating neurons (indicative of active neurotransmission / AP generation), as defined by more than 2 peaks/minute >1.08. AMA treated cells show a decrease over time.


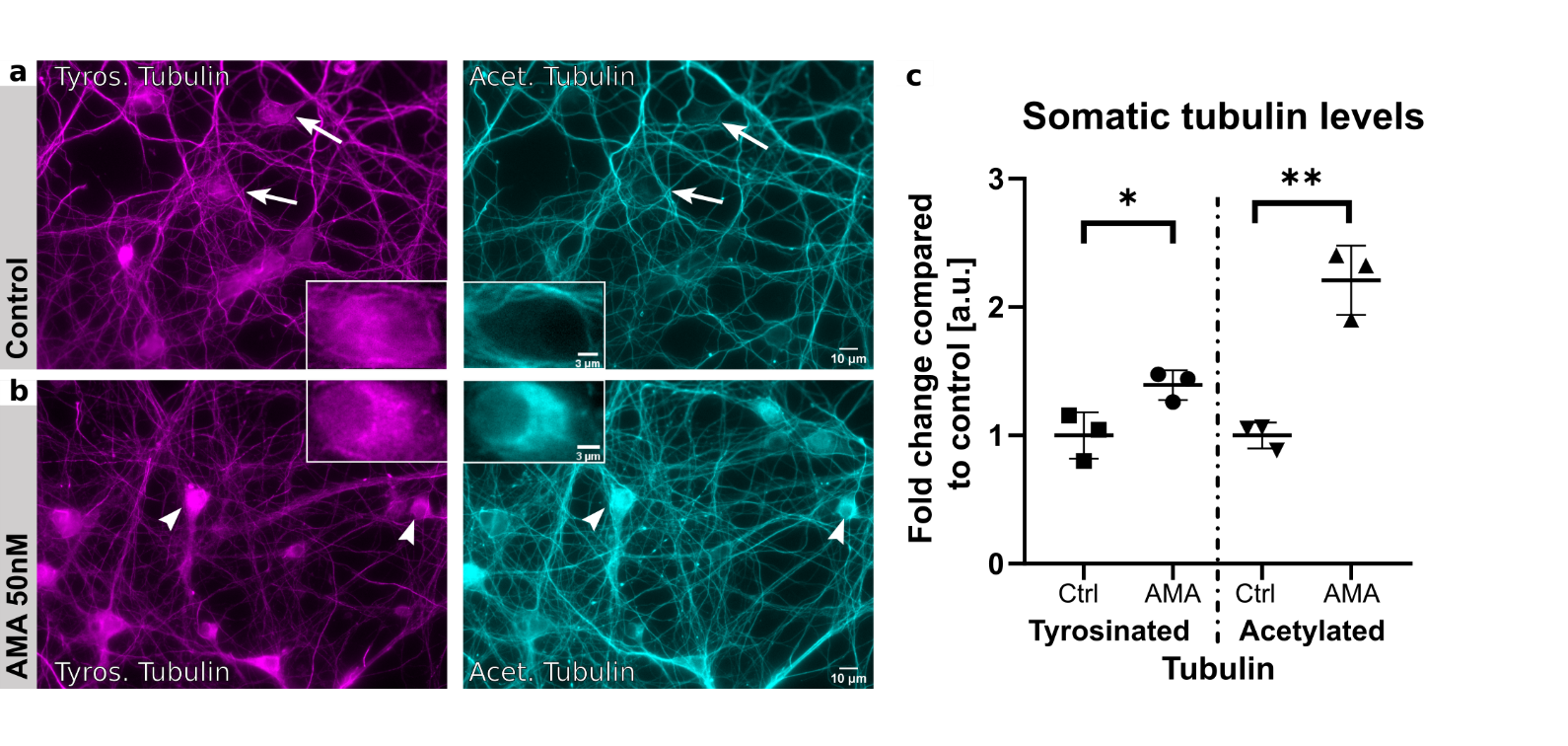


**Suppl. 4 Mitochondria dysfunction results in impaired microtubule dynamics, as assayed by microtubule PTMs**Immunofluorescence staining of MPN (div11-12) treated for 2 hours with (a) control, (b) 50nM Antimycin (AMA) for tyrosinated tubulin (left panels) and acetylated tubulin (right panels), representative overview epifluorescence images, inserts show representative magnifications of cell bodies.
a: Arrows show typical distribution of tubulins in the soma of neurons, with tyrosinated tubulin more in the soma and acetylated tubulin mostly present in the neurites.
b: Arrowheads show accumulation of acetylated tubulin, and slight increase of tyrosinated tubulin in the soma after treatment with AMA.
c: Quantification of fluorescence-intensity in the soma, arithmetic mean of 3 biological replicates with standard deviation (SD), as fold-change to control. Tyrosinated_Control_: Mean=1.0; Tyrosinated_AMA_: Mean=1.393; Acetylated_Control_: Mean=1.0; Acetylated_AMA_:Mean=2.209; all n>70 per replicate. Nested one-way ANOVA with Sidak’s correction for multiple comparisons, Tyrosinated: *, p=0.0227, Acetylated: ** p=0.0056, significance level p<0.05.


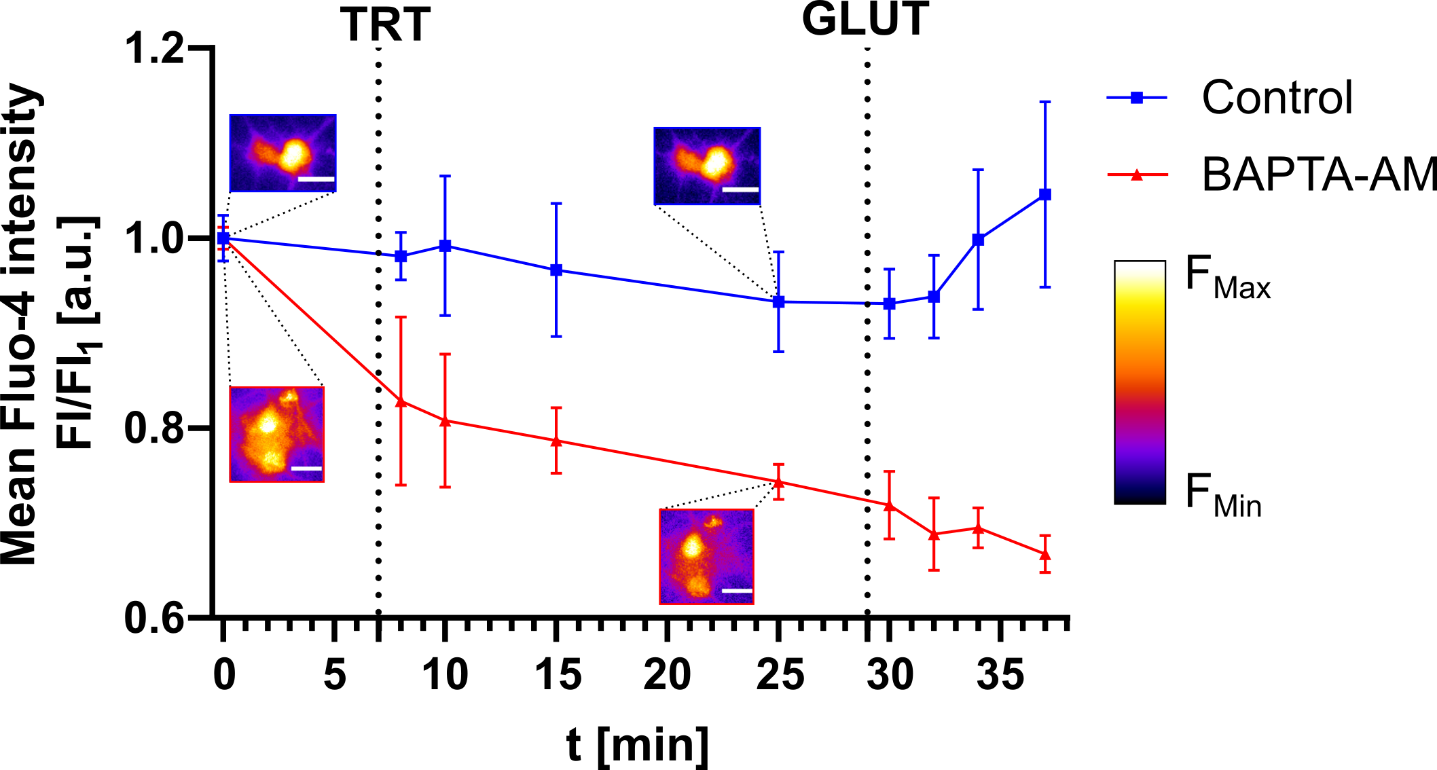


**Suppl. 5: 5µM BAPTA-AM lowers calcium levels in differentiated neurons**iPSC-derived neurons (DIV18-20) were prepared for calcium imaging (see methods) recorded at baselevel, then treated (TRT) after 7 minutes with vehicle (blue) or 5µM BAPTA-AM (red) and imaged in the same time intervals. After 29 minutes, 10µM glutamate was added (GLUT) to raise cytosolic calcium levels.
Corresponding graph showing mean fluorescence intensity of all cells in the field of view (n>60) with subtracted background over 1 minute at each timepoint, normalized to FI at t=0 and SD, 2 independent experiments per treatment. Inserts show representative, pseudocolored images of Fluo-4 intensity for control (top) and BAPTA-AM (bottom) at baseline and after 25 minutes, scale bar 20µm.
Note that vehicle treated cells maintain cytosolic calcium levels for 30mins, and respond to glutamate stimulation, while BAPTA-AM-treated cells show gradual reduction of intracellular calcium levels, which persists also under stimulating conditions.
